# Circular RNA circPRKCI contributes to malignant progression of T-cell acute lymphoblastic leukemia by modulating miR-20a-5p/SOX4 axis

**DOI:** 10.18632/aging.203647

**Published:** 2021-10-25

**Authors:** Yan Zheng, Ben Niu, Weihua Zhang, Xingli Ru, Ying Gao, Chuancui Li, Xifeng Wu

**Affiliations:** 1 Shaanxi Provincial People’s Hospital, Xi’an, Shaanxi Province, China; 2 Department of Hematology, Jinan People’s Hospital Affiliated to Shandong First Medical University, Jinan, Shandong, China

**Keywords:** T-ALL, circPRKCI, miR-20a-5p, SOX4, promising therapeutic strategy

## Abstract

Circular RNAs (circRNAs) have demonstrated critical roles in the development of cancers. This study aimed to explore the function of circular RNA circPRKCI/miR-20a-5p/SOX4 axis in acute lymphoblastic leukemia (ALL). Our data showed that the expression of circPRKCI and SOX4 was enhanced while the expression of miR-20a-5p was reduced in the clinical T-ALL samples. The expression of miR-20a-5p was negatively associated with circPRKCI and SOX4 in the T-ALL patients and the expression of circPRKCI was positive correlated with SOX4 in the T-ALL patients. Functionally, the silencing of circPRKCI suppressed the viability of T-ALL cells. Conversely, the knockdown of circPRKCI promoted the apoptosis of T-ALL cells. The levels of cleaved PARP and cleaved caspase3 were induced by the depletion of circPRKCI in T-ALL cells. Mechanically, the luciferase activity of circPRKCI was significantly decreased in T-ALL cells after the treatment of miR-20a-5p mimic. Meanwhile, the silencing of circPRKCI promoted the expression of miR-20a-5p in T-ALL cells, implying that circPRKCI serves as a competitive endogenous RNAs (ceRNA) of miR-20a-5p. We validated that the treatment of miR-20a-5p mimic inhibited the viability of T-ALL cells. MiR-20a-5p mimic enhanced the apoptosis of T-ALL cells. The expression of cleaved PARP and cleaved caspase3 was increased by miR-20a-5p mimic in the cells. In summarization, we concluded that circular RNA circPRKCI contributed to malignant progression of T-cell acute lymphoblastic leukemia by modulating miR-20a-5p/SOX4 axis. Targeting circPRKCI may serve as a promising therapeutic strategy of T-ALL.

## INTRODUCTION

Acute lymphoblastic leukemia (ALL) is the most frequently occurred malignancy in children, among which T-cell ALL (T-ALL) accounts for almost 20% of all newly diagnosed ALL cases both in children and adults [1]. Clinically, T-ALL is featured by uncontrolled generation of malignant precursor cells of the T lymphoid in the thymus and bone marrow [2]. During the process of T-ALL, leukocytosis onset and infiltrated into lymph nodes as well as other organs including central nervous system, and mediastinal mass derived from thymus would appear [3]. Current treatments for T-ALL mainly involve the transplantation of hematopoietic cells and the combination of chemical reagents for refractory diseases [4]. Although less deadly than B lymphoblastic leukemia (B-ALL) and the survival ratio is steadily improving, the relapse and cure of T-ALL are still major problems in clinical medicine and requires intensive efforts.

Circular RNAs (circRNAs) are a form of non-coding RNAs that derived from precursor RNAs, which are highly conserved and abundant [5, 6]. Unlike the usual linear structure of RNAs, circRNAs are covalently closed RNAs, which confers them high stability in physical system, comparing with other noncoding RNAs [6]. Numerous studies have proposed abnormal levels of circRNAs and their remarkable functions in various diseases, especially in cancers [7]. CircPRKCI is a newly found circRNA that derived from the 3q26.2 locus [8]. Qiu and colleagues disclosed the overexpression of circPRKCI in lung tumor tissues, which was correlated with activated tumor initiation and progression of lung cancer [8]. Silencing of circPRKCI suppressed the growth of esophageal cancer and enhanced sensitivity of cell to radiation through PARP signaling [9]. However, the function of circPRKCI in T-ALL has not been disclosed yet. Studies showed that circRNAs affected gene expression through acting as sponges of microRNAs (miRNAs) [6]. MicroRNAs (miRNAs) are a form of short length noncoding RNAs with about 20 nucleotides [10]. MiRNAs act as tumor suppressors or activators through interacting with the 3’UTR areas of specific mRNAs to suppress gene translation, which consequently causes the dysregulation of cellular process including growth and metastasis of cancer cells [10]. For example, miR-20a-5p suppressed chemoresistance in osteosarcoma, and impeded metastasis of hepatocellular carcinoma [11, 12]. A recent study also suggested that miR-20a-5p may be a potential therapeutic target for acute myeloid leukemia (AML) [13]. Its role in T-ALL is yet to be defined.

SOX4 is a critical transcriptional factor that participates in regulation of cell stemness, proliferation, differentiation, through regulating various cancer-promoting signaling pathways such as Wnt, TGFβ and PI3K signaling [14]. The amplification of SOX4 gene is frequently found in multiple malignancies and proposed as an oncogene [14]. Studies demonstrated that SOX4 regulated transcription of CXCL13 in human T helper cells in inflammatory condition [15]. Previous research proposed SOX4 as an important participant in adult T-cell leukemia [16]. In this study, we exposed the promoting role of circPRKCI in T-ALL, and deciphered the downstream miR-20a-5p/SOX4 regulatory axis. Our findings may provide new evidences to explore effective therapeutic approaches for T-ALL.

## RESULTS

### The expression levels of circPRKCI are positive correlated with SOX4 and are negative correlated with miR-20a-5p in T-ALL samples

**Figure 1:**
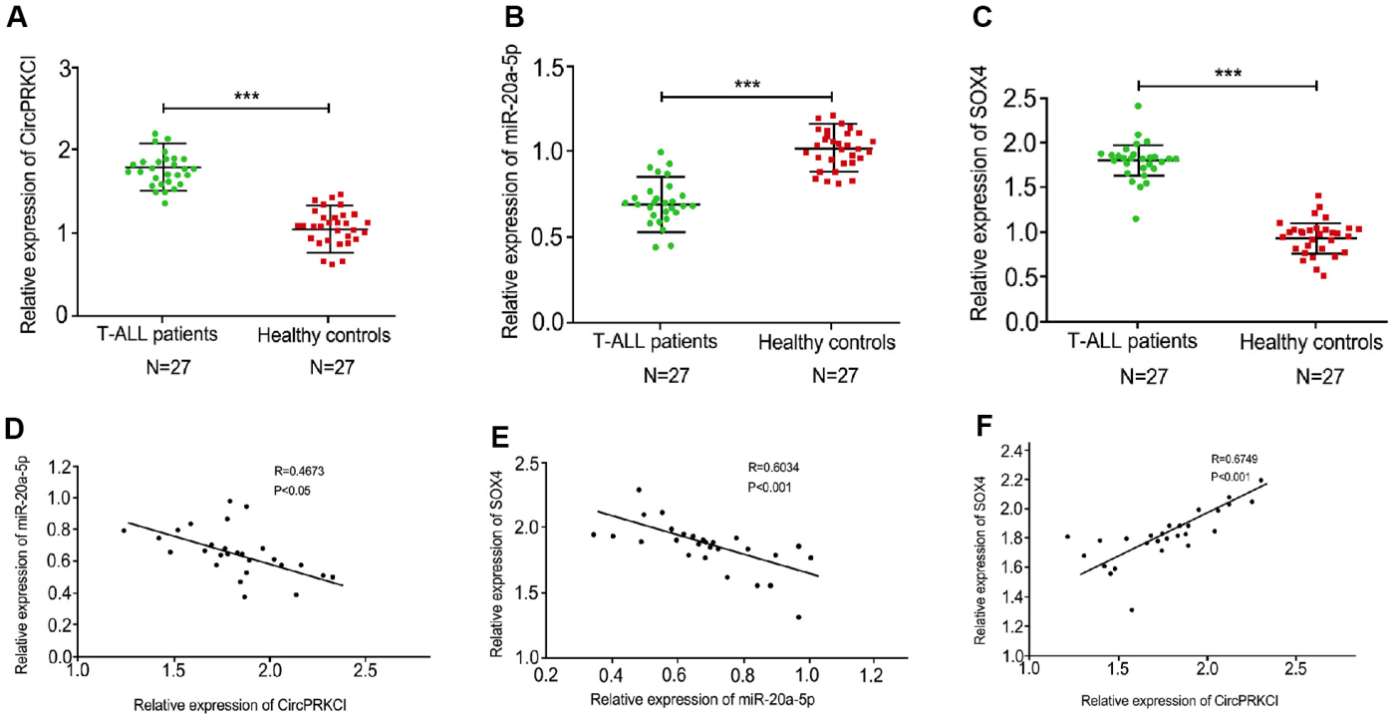
**The expression levels of circPRKCI are positive correlated with SOX4 and are negative correlated with miR-20a-5p in T-ALL samples.** (**A**, **B**) Levels of circPRKCI (**A**), miR-20a-5 (**B**) and SOX4 (**C**) in T-ALL samples were detected by qRT-PCR experiment. (**D**) The correlation of circPRKCI with miR-20a-5p was analyzed using qRT-PCR experiment in T-ALL samples. (**E**) The correlation of SOX4 with miR-20a-5p was analyzed using qRT-PCR experiment in T-ALL samples. (**F**) The correlation of SOX4 with circPRKCI was analyzed using qRT-PCR experiment in T-ALL samples. ***p < 0.001.

To determine the correlation of circPRKCI, miR-20a-5p, and SOX4 in the development of T-ALL, we evaluated the expression levels of circPRKCI, miR-20a-5p, and SOX4 in clinical T-ALL samples. Significantly, the expression of circPRKCI was enhanced while the expression of miR-20a-5p was reduced in the clinical T-ALL samples (Figure 1A, 1B). In addition, the expression of SOX4 was also induced in the clinical T-ALL patients relative to the healthy controls (Figure 1C). Moreover, the expression of miR-20a-5p was negatively associated with circPRKCI and SOX4 in the T-ALL patients (Figure 1D, 1E). Importantly, the expression of circPRKCI was positive correlated with SOX4 in the T-ALL patients (Figure 1F).

### CircPRKCI knockdown suppresses T-ALL cell survival *in vitro*

**Figure 2:**
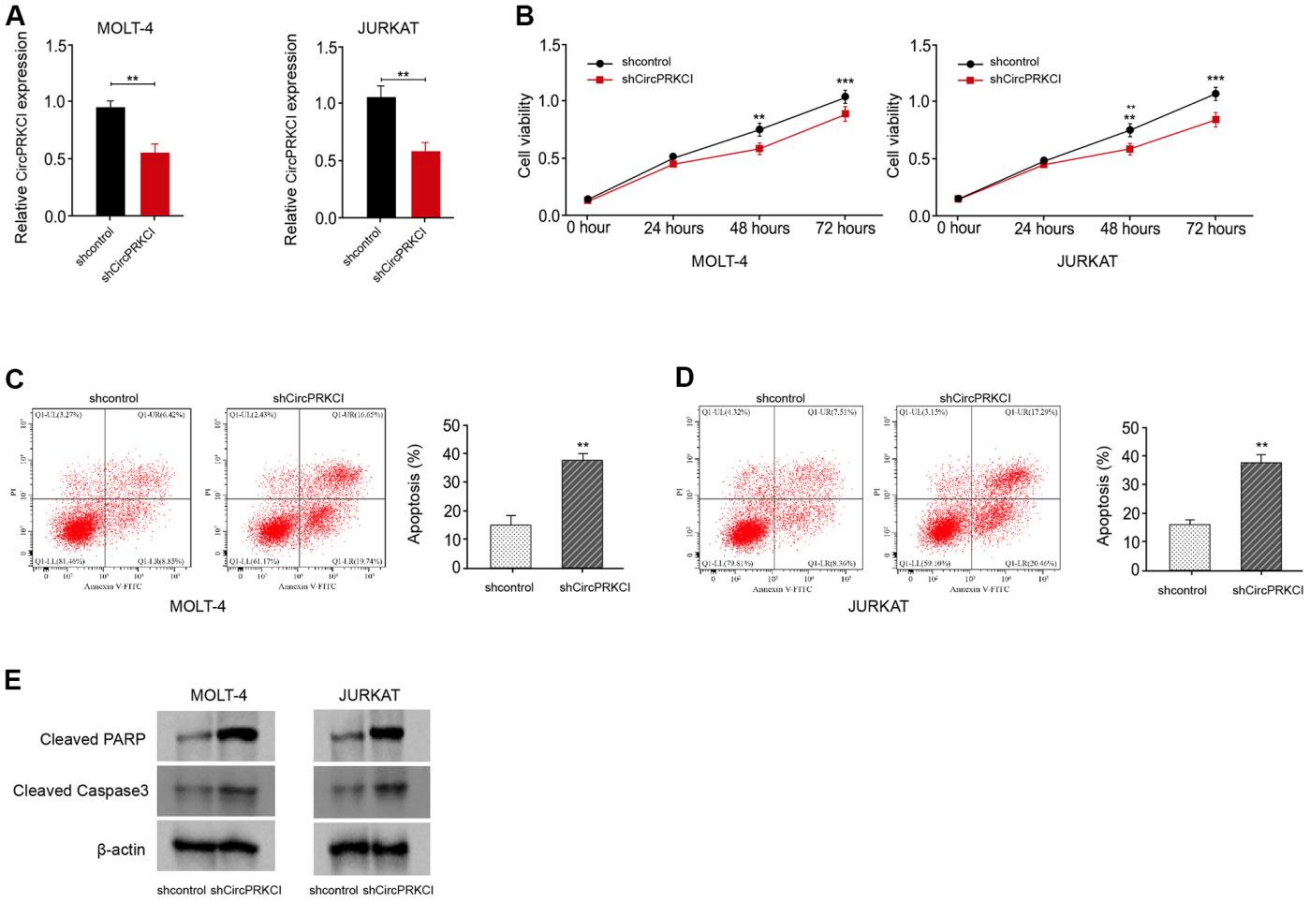
**CircPRKCI knockdown suppresses T-ALL cell survival *in vitro*.** (**A**–**E**) MOLT-4 and JURKAT cells were treated with circPRKCI shRNA. (**A**) The treatment efficacy of circPRKCI shRNA in MOLT-4 and JURKAT cells was determined by qRT-PCR assay. The cell viability (**B**) and apoptosis (**C**, **D**) were measured by CCK-8 and flow cytometry, respectively. (**E**) The expression of cleaved PARP and cleaved caspase3 was measured by Western blot analysis. **p < 0.01, ***p < 0.001.

We then explored the function of circPRKCI in the mediation of T-ALL cell survival *in vitro*. To this end, the T-ALL MOLT-4 and JURKAT cell lines were treated with circPRKCI shRNA and the treatment efficacy of circPRKCI shRNA was validated in the cells (Figure 2A). The silencing of circPRKCI using shRNA suppressed the viability of MOLT-4 and JURKAT cells (Figure 2B). Conversely, the knockdown of circPRKCI promoted the apoptosis of MOLT-4 and JURKAT cells (Figure 2C, 2D). Consistently, the levels of cleaved PARP and cleaved caspase3 were induced by the depletion of circPRKCI in MOLT-4 and JURKAT cells (Figure 2E), indicating that CircPRKCI knockdown suppresses T-ALL cell survival *in vitro*.

### CircPRKCI serves as a ceRNA of miR-20a-5p in T-ALL cells

**Figure 3:**
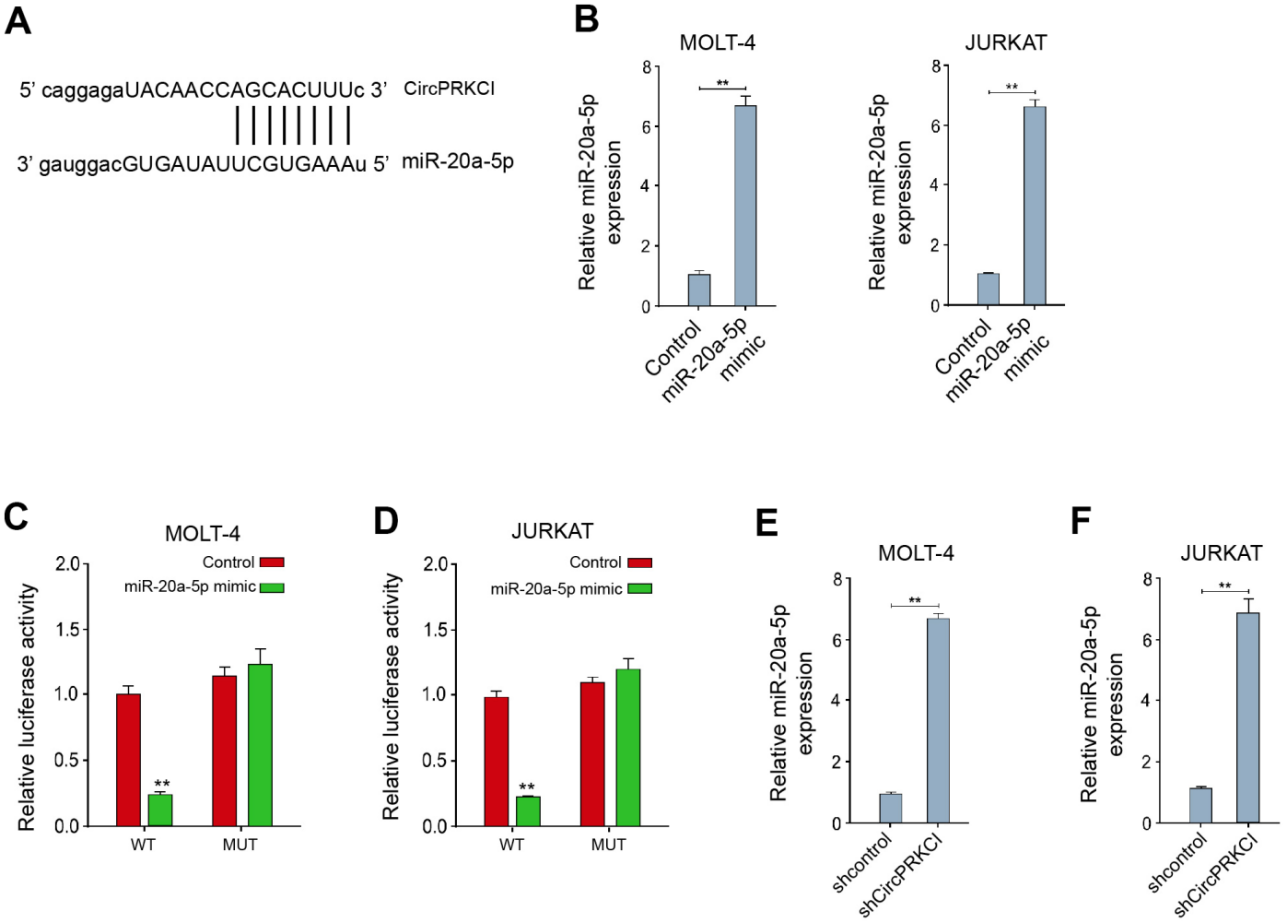
**CircPRKCI serves as a ceRNA of miR-20a-5p in T-ALL cells.** (**A**) The predicted interaction site between circPRKCI and miR-20a-5p. (**B**–**D**) MOLT-4 and JURKAT cells were treated with miR-20a-5p mimic. (**B**) The treatment efficacy of miR-20a-5p mimic in MOLT-4 and JURKAT cells was determined by qRT-PCR assay. (**C**, **D**) The luciferase activity of circPRKCI was determined by dual luciferase reporter assay. (**E**, **F**) MOLT-4 and JURKAT cells were treated with circPRKCI shRNA. The expression of miR-20a-5p was examined by qRT-PCR assay. **p < 0.01.

Next, we verified the relationship of circPRKCI and miR-20a-5p. We identified the predicted binding site between circPRKCI and miR-20a-5p in the bioinformatics analysis (Figure 3A). The treatment of miR-20a-5p mimic remarkably increased miR-20a-5p expression in MOLT-4 and JURKAT cells (Figure 3B). The luciferase activity of circPRKCI was significantly decreased in MOLT-4 and JURKAT cells after the treatment of miR-20a-5p mimic (Figure 3C, 3D).

Meanwhile, the silencing of circPRKCI using shRNA promoted the expression of miR-20a-5p in MOLT-4 and JURKAT cells (Figure 3E, 3F), implying that circPRKCI serves as a ceRNA of miR-20a-5p in T-ALL cells.

### CircMRPS35 regulates PD-L1 expression and cell growth in TNBC

### MiR-20a-5p represses T-ALL cell survival in vitro

**Figure 4:**
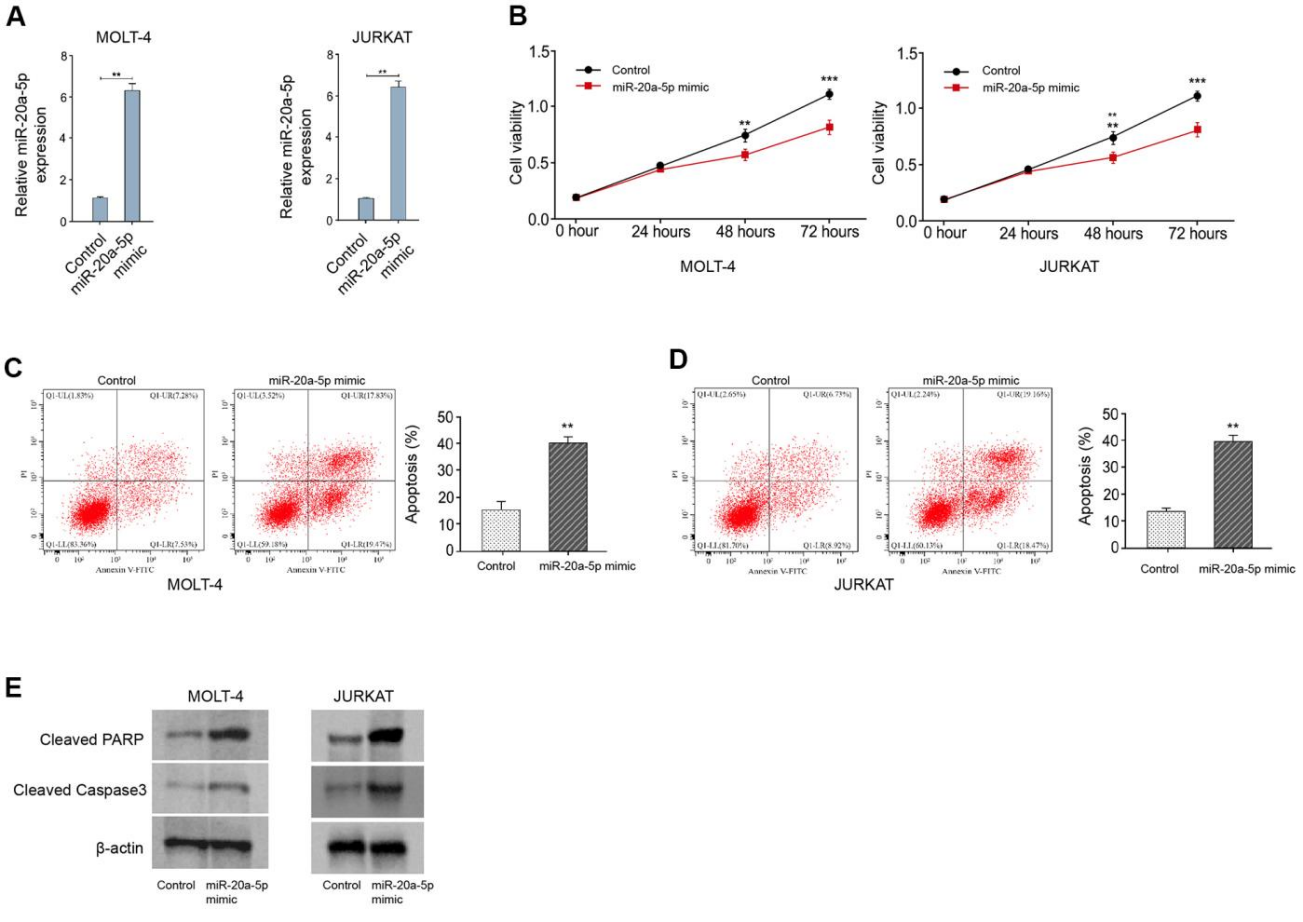
**MiR-20a-5p represses T-ALL cell survival *in vitro*.** (**A**–**E**) MOLT-4 and JURKAT cells were treated with miR-20a-5p mimic. (**A**) The treatment efficacy of miR-20a-5p mimic in MOLT-4 and JURKAT cells was determined by qRT-PCR assay. The cell viability (**B**) and apoptosis (**C**, **D**) were measured by CCK-8 and flow cytometry, respectively. (**E**) The expression of cleaved PARP and cleaved caspase3 was measured by Western blot analysis. **p < 0.01, ***p < 0.001.

Then, we validated the function of miR-20a-5p in the modulation of T-ALL cell survival *in vitro*. For this purpose, the T-ALL MOLT-4 and JURKAT cell lines were treated with miR-20a-5p mimic and the treatment efficacy of miR-20a-5p mimic was confirmed in the cells (Figure 4A). The treatment of miR-20a-5p mimic inhibited the viability of MOLT-4 and JURKAT cells (Figure 4B). Reversely, miR-20a-5p mimic enhanced the apoptosis of MOLT-4 and JURKAT cells (Figure 4C, 4D). Similarly, the levels of cleaved PARP and cleaved caspase3 were increased by miR-20a-5p mimic in MOLT-4 and JURKAT cells (Figure 4E), indicating that miR-20a-5p represses T-ALL cell survival *in vitro*.

### MiR-20a-5p can target SOX4 in T-ALL cells

**Figure 5:**
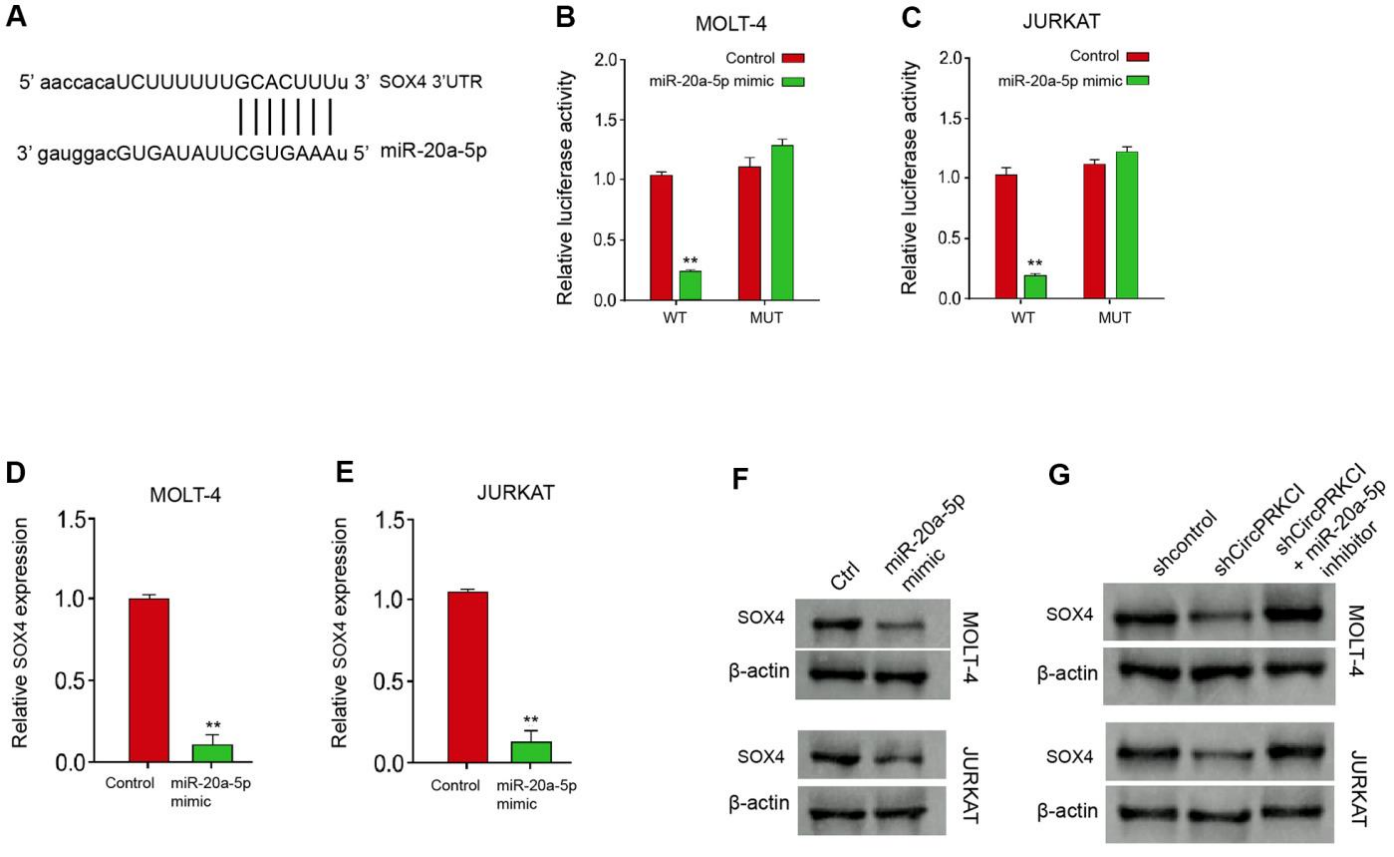
**MiR-20a-5p can target SOX4 in T-ALL cells.** (**A**) The predicted interaction site between circPRKCI and miR-20a-5p. (**B**–**F**) MOLT-4 and JURKAT cells were treated with miR-20a-5p mimic. (**B**, **C**) The treatment efficacy of miR-20a-5p mimic in MOLT-4 and JURKAT cells was determined by qRT-PCR assay. (**D**, **E**) The luciferase activity of SOX4 was determined by dual luciferase reporter assay. (**F**) The expression of SOX4 was determined by Western blot analysis. (**G**) MOLT-4 and JURKAT cells were treated with circPRKCI shRNA or co-treated with miR-20a-5p inhibitor. The expression of SOX4 was analyzed by Western blot analysis. **p < 0.01.

We then investigated the interaction of miR-20a-5p and SOX4. We found the predicted binding site between SOX4 and miR-20a-5p in the bioinformatics analysis (Figure 5A). The luciferase activity of SOX4 was significantly suppressed in MOLT-4 and JURKAT cells after the treatment of miR-20a-5p mimic (Figure 5B, 5C). Both mRNA and protein levels of SOX4 were remarkably repressed by the treatment of miR-20a-5p mimic in MOLT-4 and JURKAT cells (Figure 5D–5F). Crucially, the expression of SOX4 was decreased in MOLT-4 and JURKAT cells after the depletion of circPRKCI using shRNA, while the inhibitor of miR-20a-5p could rescue the decrease in the cells (Figure 5G).

### SOX4 knockdown suppresses T-ALL cell survival in vitro

**Figure 6:**
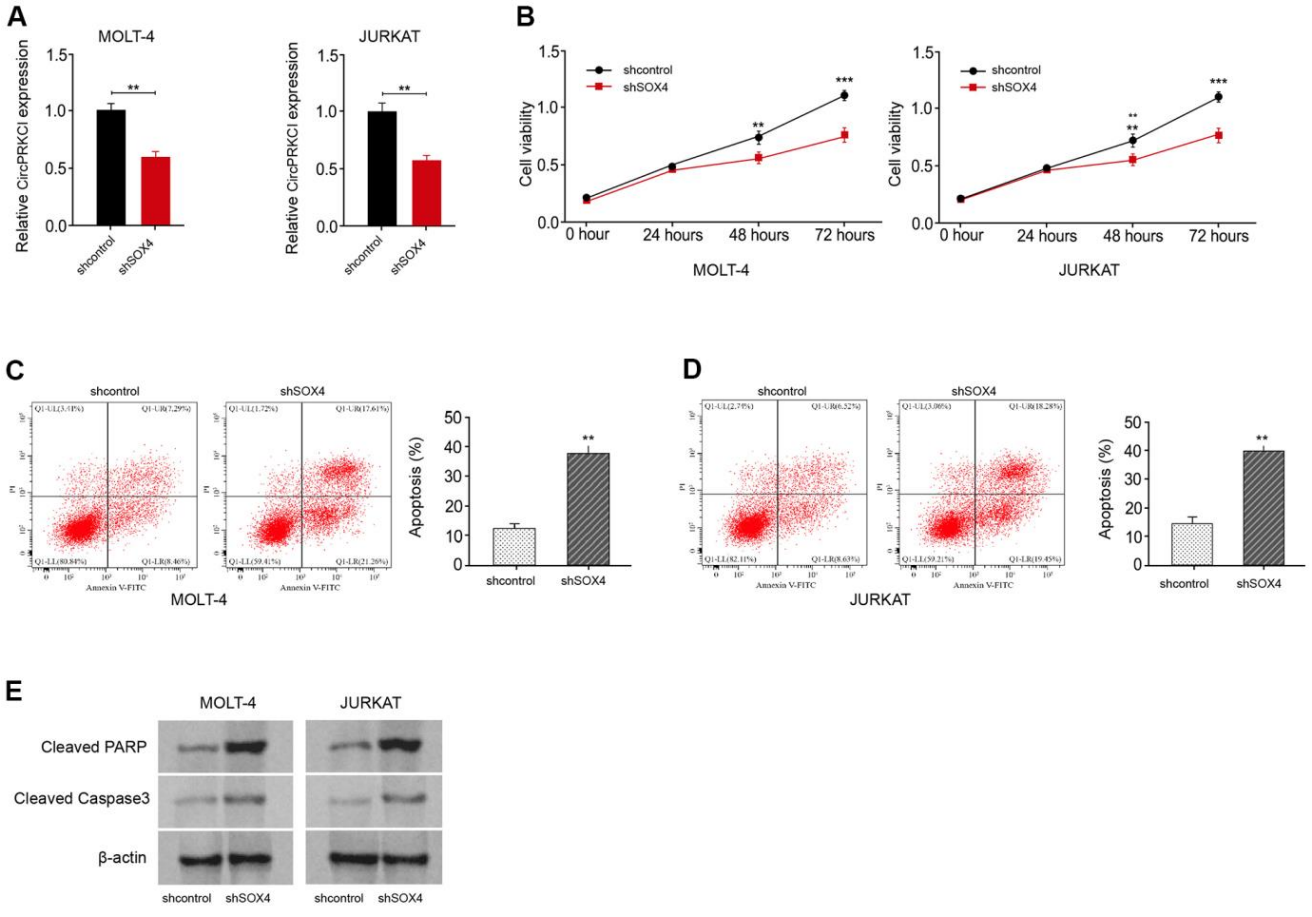
**SOX4 knockdown suppresses T-ALL cell survival *in vitro*.** (**A**–**E**) MOLT-4 and JURKAT cells were treated with SOX4 shRNA. (**A**) The treatment efficacy of SOX4 shRNA in MOLT-4 and JURKAT cells was determined by qRT-PCR assay. The cell viability (**B**) and apoptosis (**C**, **D**) were measured by CCK-8 and flow cytometry, respectively. (**E**) The expression of cleaved PARP and cleaved caspase3 was measured by Western blot analysis. **p < 0.01, ***p < 0.001.

Next, we further validated the function of SOX4 in T-ALL cell survival *in vitro*. The depletion efficacy of SOX4 shRNA was verified in MOLT-4 and JURKAT cells (Figure 6A). The knockdown of SOX4 using shRNA decreased the viability of MOLT-4 and JURKAT cells (Figure 6B). Meanwhile, the silencing of SOX4 induced the apoptosis of MOLT-4 and JURKAT cells (Figure 6C, 6D). In addition, the levels of cleaved PARP and cleaved caspase3 were promoted by the depletion of SOX4 in MOLT-4 and JURKAT cells (Figure 6E), indicating that SOX4 knockdown suppresses T-ALL cell survival *in vitro*.

### CircPRKCI/ miR-20a-5p/SOX4 axis regulates T-ALL cell survival in vitro

**Figure 7:**
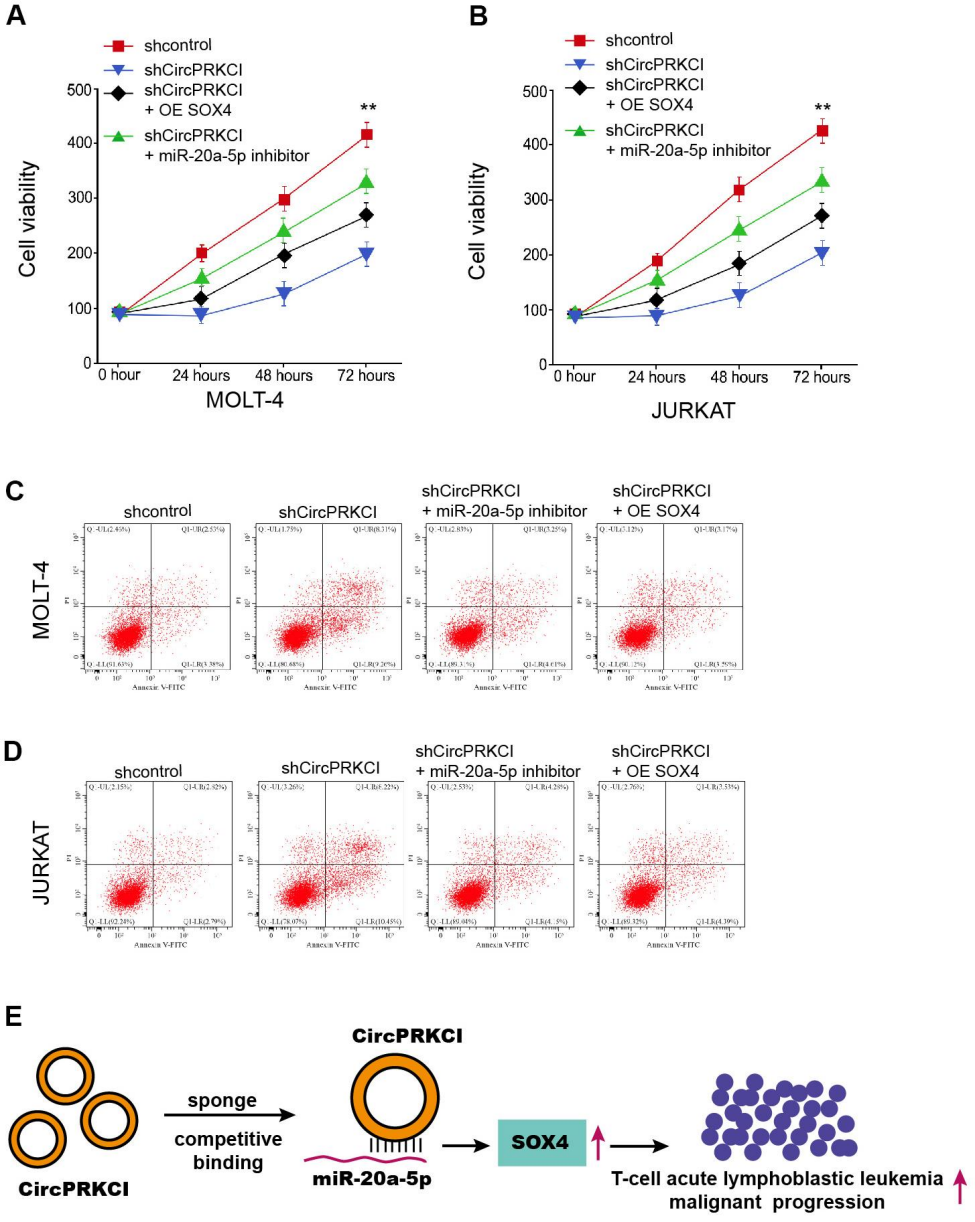
**CircPRKCI/miR-20a-5p/SOX4 axis regulates T-ALL cell survival *in vitro*.** (**A**–**D**) MOLT-4 and JURKAT cells were treated with circPRKCI shRNA, or co-treated miR-20a-5p inhibitor or SOX4 vectors. (**A**, **B**) The cell viability was detected by CCK-8. (**C**, **D**) The cell apoptosis was measured by flow cytometry. **p < 0.01 (**E**).

Then, we tried to assess the correlation of circPRKCI, miR-20a-5p, and SOX4 in the modulation of T-ALL cell survival *in vitro.* As expected, the MOLT-4 and JURKAT cell viability was reduced by circPRKCI silencing, while the overexpression of SOX4 or suppression of miR-20a-5p rescued the reduced viability in MOLT-4 and JURKAT cells (Figure 7A, 7B). Besides, the knockdown of circPRKCI enhanced MOLT-4 and JURKAT cell apoptosis, and SOX4 re-expression or miR-20a-5p inhibition could reverse this enhancement (Figure 7C, 7D).

## DISCUSSION

ALL serves as a prevalent and severe malignancies, among which T-ALL is a major type of ALL and the molecular mechanisms of the development of T-ALL are poorly investigated. CircRNAs have demonstrated critical roles in different processes, such as proliferation, invasion, apoptosis, autophagy, and ferroptosis, during the cancer progression. However, the function of circRNA circPRKCI in the modulation of T-ALL remains unreported. In the present work, we uncovered the important role of circPRKCI in T-ALL cells.

The previous investigations have reported the function of circRNAs in T-ALL development. The enhancement of circ-0000745 enhanced cell viability by the activation of ERK signaling in ALL [17]. Circular RNA circRNA_0000094 serves as a sponge of microRNA-223-3p to enhance F-box expression and restrains T-ALL progression [18]. Meanwhile, it has been found that circPRKCI is a ceRNA to enhance AKT3 expression in esophageal squamous cell carcinoma via sponging miR-3680-3p [19]. CircPRKCI attenuates lipopolysaccharide-stimulated injury of HK2 cells by the up-regulation of ZEB2 by sponging miR-545 [20]. CircPRKCI depletion contributes to radiosensitivity and represses progression of esophageal cancer by the regulation of miR-186-5p/PARP9 axis [9]. CircPRKCI enhances progression of glioma cells via suppressing microRNA-545 [21]. In our research, we observed that the expression of circPRKCI was enhanced in the clinical T-ALL samples. The silencing of circPRKCI suppressed the viability of T-ALL cells. Conversely, the knockdown of circPRKCI promoted the apoptosis of T-ALL cells. The levels of cleaved PARP and cleaved caspase3 were induced by the depletion of circPRKCI in T-ALL cells. These data reveal the crucial function of circPRKCI in promoting cell proliferation of T-ALL, indicating the innovative role of circPRKCI in T-ALL cells and present a new experimental evidence of the crucial impact of circRNAs on T-ALL development. As the progression of T-ALL is complicated and correlated with the immune response, the function of circPRKCI in immunity during T-ALL progression should be explored in future studies.

In recent years, increasing number of studies have identified the function of miRNAs in T-ALL. MiR-101 inhibits T-ALL by modulating CXCR7/STAT3 signaling [22]. MiR-22-3p represses cancer progression in T-ALL [23].

miR-141-3p/TRAF5 axis enhances the progression of T-ALL [24]. In addition, it has been reported that mir-20a-5p regulates progressions of gastric cancer by modulating AKT signaling [25]. MiR-20a-5p contributes to migration and proliferation of pulmonary artery smooth muscle cells by inhibiting ABCA1 [25]. MiR-20a-5p reduces cell proliferation of neuroblastoma by targeting autophagy [26]. Moreover, microRNA-204 suppresses in T-ALL cell proliferation via the down-regulation of SOX4 [27]. In the present work, we identified that the expression of miR-20a-5p was reduced and SOX4 expression was induced in the clinical T-ALL samples. The expression of miR-20a-5p was negatively associated with circPRKCI and SOX4 in the T-ALL patients and the expression of circPRKCI was positive correlated with SOX4 in the T-ALL patients. CircPRKCI served as a ceRNA of miR-20a-5p and miR-20a-5p targeted SOX4 in T-ALL cells, in which circPRKCI sponged miR-20a-5p to promote SOX4 expression. These data elucidate an unreported mechanism by which circPRKCI enhances SOX4 expression by sponging miR-20a-5p in T-ALL cells. Meanwhile, we validated that miR-20a-5p or SOX4 knockdown suppressed T-ALL cell survival *in vitro*. The overexpression of SOX4 or miR-20a-5p repression by inhibitor could reverse the effect of circPRKCI depletion of cell viability and apoptosis of T-ALL. These data imply that circPRKCI promotes T-ALL progression at least partly by the miR-20a-5p/SOX4 axis, indicating a new function of miR-20a-5p in T-ALL. The effect and correlation of circPRKCI/ miR-20a-5p/SOX4 axis in T-ALL should be validated in mouse model *in vivo*. Meanwhile, other mechanisms of circPRKCI and miR-20a-5p need to be explored in future.

## CONCLUSIONS

To summarize, we concluded that circular RNA circPRKCI contributed to malignant progression of T-cell acute lymphoblastic leukemia by modulating miR-20a-5p/SOX4 axis (Figure 7E). Targeting circPRKCI may serve as a promising therapeutic strategy of T-ALL.

## MATERIALS AND METHODS

### Clinical specimen

The bone marrow tissues were collected from patients newly diagnosed as T-ALL (n=27) who hospitalized in our hospital from February 2017 to July 2020, and healthy donors as control (n=27). All participants have signed the informed consent. The experiment was approved by the Clinical Ethics Committee of Shaanxi Provincial People’s Hospital. Patients were selected by following criteria: patients with arrhythmia, heart dysfunction, hepatic or renal dysfunction, lung dysfunction and other tumor history were excluded. Flowcytometry analysis identified positive staining of T cell biomarkers (CD2, CD3, CD4, CD8) and stem cell biomarker (CD34 and CD38), and negative staining of myeloid biomarkers such as CD13, CD15, CD33 and CD117 were diagnosed as T-ALL patients. The T-ALL patients included 9 females and 18 males, averagely aged 32-years old. The healthy donors included 15 females and 12 males, averagely aged 30-years old.

### Cell culture and transfection

The human T-ALL cell lines MOLT-4 and JURKAT were purchased from American Type Cell Collection (ATCC, USA), and cultured in RPMI-1640 medium which contains 10% fetal bovine serum (FBS, Gibco, USA) and 1% penicillin/streptomycin mixture (Thermo Fisher, USA). Cells were maintained in humidified 5% CO_2_ atmosphere at 37° C. For cell transfection, the short hairpin RNA (shRNA) targeting CircPRKCI (shCircPRKCI), shRNA targeting SOX4 (shSOX4), shRNA negative control (shNC), SOX4 overexpressing plasmid (pcDNA-SOX4) and control plasmid (pcDNA-NC), miR-20a-5p mimics and inhibitors were purchased from GenePharma (China). The transfection reagent Lipofectamine 2000 was obtained from Invitrogen (USA) and used for cell transfection according to manufacturer’s instruction. The dose of oligonucleotides used for transfection was 50 nM.

### CCK-8 assay

MOLT-4 and JURKAT cells were placed in a 96-well plate (3000/well) after transfection with indicated oligonucleotides. After incubation for 0, 24, 48, and 72 hours, Cell Counting Kit-8 (CCK-8, Thermo Fisher) reagent was added into each well, and cultured for 1 hour. Subsequently, optical density of 450 nm was detected by a microplate detector (BD Biosciences, USA).

### Apoptosis

Cell apoptosis was determined by a FITC-Annexin V/PI Apoptosis detection kit (Thermo Fisher) following manufacturer’s protocol. In brief, MOLT-4 and JURKAT cells were digested and suspended in binding buffer after cell transfection. Then FITC-Annexin V (5 μL/well) and PI were added into each well, and hatched at room temperature for 30 minutes. The apoptotic cell portion was detected by a flow cytometer (BD Biosciences).

### RNA quantification

Total RNA was extracted from patient samples, MOLT-4 and JURKAT cells via TRIzol reagent (Invitrogen) in accordance with manufacturer’s instruction. The complementary DNA was synthesized from total RNA (1 μg) in a total volume of 20 μl by using a High-Capacity cDNA reverse transcription kits (Thermo Fisher). The reaction mixture was incubated at 25° C for 5 min for priming, then at 42° C for 30 min for reverse transcription, and finally at 85° C for 5 min for reverse transcriptase inactivation. The complementary DNA (cDNA) was stored at −20° C until further use. Then the samples were subjected to quantification by SYBR Green Master Mix (Thermo Fisher). 400nM specific sense primer, 400 nM specific antisense primer, RNase/DNase-free water, and cDNA template (previously isolated and reverse-transcribed RNA of 1 ng/well) up to final volume of 10 μl. Amplifications were performed starting with a 30 sec enzyme activation at 95° C, followed by 40 cycles of denaturation at 95° C for 5 sec, and then annealing/extension at 60° C for 30 sec. At the end of each run a melting curve analysis was performed from 65–95° C. All samples and negative controls were amplified in triplicate, and the obtained mean value was then used for further analysis. Cycle of quantification (Cq) values of >35 was excluded from further mathematical calculations. Relative gene expression was normalized to GAPDH or U6 and calculated by 2^−ΔΔCt^ method. The experiments were repeated at least for 3 times. Primer sequences were as following:

circPRKCI, F, 5’-ATTCAGGGACACCCGTTCTT-3’, R, 5’-CTCTTCAGAACACTTGCAGCTT-3’;

miR-20a-5p, F, 5’-UAAAGUGCUUAUAGUGCAGGUAG-3′, R, 5′-CUACCUGCACUAUAAGCACUUUA-3′;

SOX4, F, 5’-AGCGACAAGATCCCTTTCATTC-3’, R, 5’-CGTTGCCGGACTTCACCTT-3’;

U6, F, 5′-CTCGCTTCGGCAGCACATATACT-3′, R, 5′-ACGCTTCACGAATTTGCGTGTC-3′;

GAPDH, F, 5′-CGGAGTCAACGGATTTGGTCGTAT -3′, R, 5′-AGCCTTCTCCATGGTGGT GAAGAC -3′.

### Western blotting

Cleaved PARP, cleaved Caspase3, β-actin, SOX4, Total proteins were extracted from patient samples, MOLT-4 and JURKAT cells via RIPA lysis buffer (Invitrogen), and quantified by using a BCA kit (Beyotime, China). The proteins were separated by sodium dodecylsul-fate polyacrylamide gel electrophoresis (SDS-PAGE) and shifted onto a PVDF membrane (Millipore, USA). The membranes were then blocked in 5% defatted milk, followed by incubation with the primary antibodies against Cleaved PARP (1:1000, Proteintech, China), cleaved Caspase3 (1:1000, Proteintech), β-actin (1:1000, Proteintech) and SOX4 (1:1000, Proteintech) at 4° C overnight. Next day, the membranes were hatched in the secondary anti-rabbit antibody. The chemiluminescence was developed with ECL kit (Thermo Fisher).

### Dual luciferase reporter assay

Binding sequences of miR-20a-5p to circPRKCI and SOX4 3’UTR were predicted via bioinformatics analysis. wild type reporter gene plasmids (WT) and mutated reporter gene plasmids (MUT) of circPRKCI and SOX4 were constructed by inserting corresponding sequences into pmirGLO reporter plasmid (Promega, USA). MOLT-4 and JURKAT cells were seeded in 12-well plates and transfected with MUT or WT and miR-20a-5p mimics or NC. The luciferase activity was detected by a Dual-Luciferase Reporter Assay System (Promega, USA).

### Statistics

Data in this work were presented as mean ± SD of three replicates. SPSS software (Version 22.0) was adopted for comparison between two or more groups via Student’s t-test or one-way ANOVA method. P < 0.05 was taken as statistically significant.
